# Discovery of ER-localized sugar transporters for cellulase production with *lac1* being essential

**DOI:** 10.1186/s13068-022-02230-x

**Published:** 2022-11-29

**Authors:** Haiyan Wang, Ai-Ping Pang, Wei Wang, Bingzhi Li, Chengcheng Li, Fu-Gen Wu, Fengming Lin

**Affiliations:** 1 State Key Laboratory of Bioelectronics, School of Biological Science and Medical Engineering, Southeast University, Nanjing, China; 2 State Key Lab of Bioreactor Engineering, New World Institute of Biotechnology, East China University of Science and Technology, Shanghai, China; 3 Key Laboratory of Systems Bioengineering (Ministry of Education), School of Chemical Engineering and Technology, Tianjin University, Tianjin, China; 4 School of Light Ind. & Food Sci. and Joint International Research Lab of Lignocellulosic Functional Materials, Nanjing Forestry University, Nanjing, 210037 China

**Keywords:** Filamentous fungi, *Trichoderma reesei*, Cellulase, Nucleotide sugar transporter, Endoplasmic reticulum

## Abstract

**Background:**

In the process of cellulose hydrolysis, carbohydrate hydrolysates are transported into cells through membrane transporters, and then affect the expression of cellulase-encoding genes. Sugar transporters play a crucial role in cellulase production in lignocellulolytic fungi, of which relatively few have been functionally validated to date and are all reported to be on cell membrane.

**Result:**

Through transcriptome analysis and qRT-PCR, three putative MFS sugar transporters GST, MFS, and LAC1 were found to display significantly higher mRNA levels in *T. reesei* grown on cellulose than on glucose. The individual deletion of these three genes compromised cellulase production and delayed sugar absorption by 24 h in *T. reesei*. Nevertheless, they transported pretty low level of sugars, including galactose, lactose, and mannose, and did not transport glucose, when expressed in yeast system. Meanwhile, all three transporters were unexpectedly found to be intracellular, being located in endoplasmic reticulum (ER). Particularly, the knockout of *lac1* almost abolished cellulase production, and significantly inhibited biomass generation regardless of sugar types, indicating that *lac1* is essential for cellulase production and biomass formation*.* The absence of *lac1* upregulated genes involved in ribosome biogenesis, while downregulated genes in cellulase production, protein processing in ER (particularly protein glycosylation), and lipid biosynthesis. The inhibition of *lac1* deletion on the transcriptional levels of genes related to cellulase biosynthesis was restored after 72 h, but the cellulase production was still inhibited, indicating *lac1* might pose a post-transcription regulation on cellulase production that are independent on the known cellulase regulation mediated by CRT1 and XYR1.

**Conclusion:**

For the first time, intracellular sugar transporters (*mfs*, *gst*, and *lac1*) facilitating cellulase production were identified, which was distributed in ER. Their sugar transporting ability was very weak, indicating that they might be related to sugar utilization inside cells rather than the cellular sugar uptake. More importantly, sugar transporter *lac1* is first found to be essential for cellulase production and biomass formation by affecting protein processing in ER (particularly protein glycosylation) and lipid biosynthesis. The effect of LAC1 on cellulase production seems to be post-transcriptional at late stage of cellulase production, independent on the well-known cellulase regulation mediated by CRT1 and XYR1. These findings improve the understanding of intracellular sugar transporters in fungi and their important role in cellulase synthesis.

**Supplementary Information:**

The online version contains supplementary material available at 10.1186/s13068-022-02230-x.

## Background

Lignocellulose biomass can be exploited as renewable feedstock for bio-based products, which contains plant cell wall polysaccharides (cellulose, hemicellulose, pectin) and storage polysaccharides (starch and inulin) [1]. These polysaccharides need to be degraded into soluble sugars before fermentation with microorganisms, which can be achieved using carbohydrate-active enzymes (CAZyme), such as cellulase, hemicellulase, and xylanase, produced by lignocellulolytic fungi and bacteria [2–4]. During cellulase production in lignocellulolytic fungi utilizing cellulose, cellulose is degraded into mono-, di-, or oligo-saccharides that are transported into cells via a vast number of sugar transporters, thereby impacting the expression of cellulase-encoding genes [5, 6]. Some sugar transporters take crucial part in induction of cellulase expression. It is believed that the induced expression of sugar transporters might be a prerequisite for the induction of cellulase-encoding genes, by enhancing the internalization of inducible sugars into the cells [7–9]. To study the role of sugar transporters in cellulase production, comparative transcriptome and/or secretome have been performed to identify sugar transporters involved in cellulase production in *Aspergillus fumigatus* on sugarcane bagasse [10], *Trichoderma reesei* on cellulose [11], *Neurospora crassa* on avicel [12], and *Penicillium oxalicum* on cellulose [13]. However, these systematic analysis data can only provide the possible sugar transporter candidates that might play a role in cellulase production. Extensive biochemical efforts are still required to investigate the identities and functions of the identified sugar transporters. Currently, only a few sugar transporters related to cellulase synthesis in lignocellulolytic fungi have been characterized [8, 14–16], which are all reported to be distributed on cell membrane. For instance, in *N. crassa*, sugar transporters CDT-1 and CDT-2 are identified to be “transceptors”, which participate in cellulose sensing and signaling, and cellodextrin transportation [14]. In *A. nidulans*, sugar transporter CltA transports cellobiose, whereas CltB is mainly responsible for substrate sensing and signaling [15]. Wang et al*.* studied the effect of the glucose dual-affinity transport system on cellulase production in *N. crassa* [8]. It was found that the high-affinity glucose transport system mediated by glucose transporter HGT-1/-2 on cell membrane negatively affects cellulase production through the CCR activation. Despite this progress, most sugar transporters involved in fungal cellulase production still remain to be explored, let alone the intracellular sugar transporters.

*T. reesei* is the well-known saprophytic fungus in the enzyme industry for the production of biomass degrading enzymes, such as cellulases and hemicellulases, and heterologous protein production. The genome of *T. reesei* has been predicted to possess 50–100 sugar transporter genes [7, 17], only a few of them have been characterized in the literature, such as xylose transporter [18–20], L-arabinose transporter [21], and other sugar transporters [22, 23]. In particular, two MFS sugar transporters, cellulose response transporter (Crt1) and the major facilitator superfamily sugar transporter (Stp1), are involved in cellulose sensing and cellulase induction [22]. Crt1 acted as an important sensor or transceptor in cellulase gene induction [16, 24], showing high affinity toward cellobiose and lactose in the yeast system [16]. Its deletion abolished the expression of cellulase genes in *T*. *reesei* [22]. Stp1 could transport not only cellobiose but also glucose [16], whose knockout enhanced cellulase gene induction in *T. reesei.* Moreover, sugar transporter Tr69957, which can transport xylose, mannose, and cellobiose, has been reported to be associated with cellulase biosynthesis [25].

To explore the crucial sugar transporters involved in cellulase biosynthesis of lignocellulolytic fungi, comparative transcriptome analysis was performed to identify 49 sugar transporter genes to be related to cellulase production. Among them, sugar transporters MFS, GST, and LAC1 exhibited a positive impact on cellulase production as indicated by qRT-PCR and gene knockout, with LAC1 being required. Surprisingly, all these sugar transporters were intracellular, being distributing in ER. Moreover, they could only transport minus level of sugars, although they were positively involved in sugar consumption in *T. reesei.* Interestingly, the absence of *lac1* significantly inhibited biomass formation regardless the sugar types. Also, the effect of *lac1* on the transcriptome of *T. reesei* was investigated. For the first time as far as we know, ER-localized sugar transporters were discovered for cellulase production, shedding new light on the molecular mechanism of fungal cellulase biosynthesis and improving the understanding of sugar transporters in fungi.

## Results

### Putative sugar transporters involved in cellulase production in T. reesei Rut-C30 with lac1 being essential

**Fig. 1:**
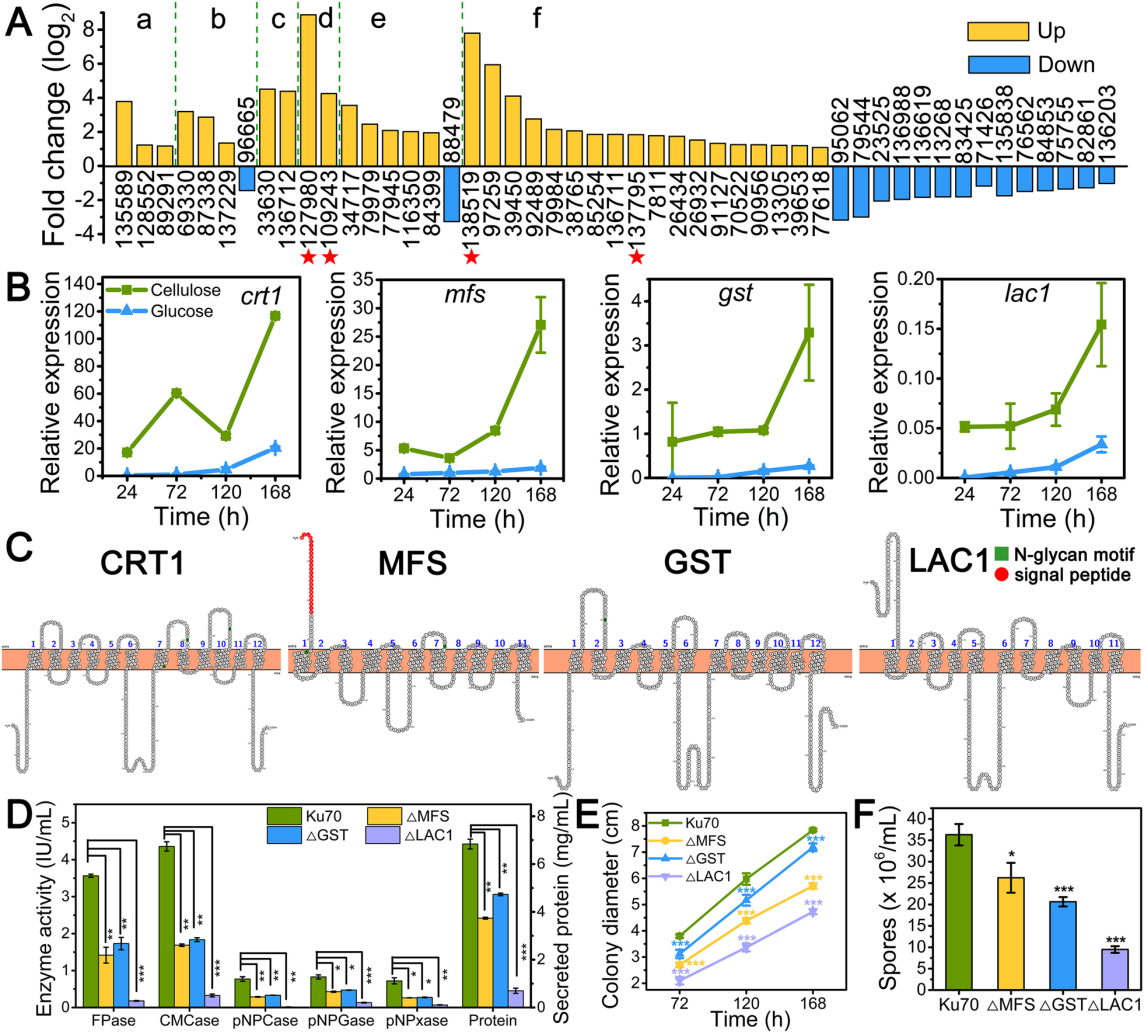
Identification of sugar transporters related to cellulase production. **A** DEGs of sugar transporters in *T. reesei* Rut-C30 grown on cellulose as compared to glucose, which are predicted to transport **a** maltose, **b** fucose, **c** xylose, **d** lactose, **e** d-galactonate, and **f** unknown sugars. The red pentagrams mark the genes studied in this research, including M419DRAFT_127980 (*lac1*), M419DRAFT_109243 (*crt1*), M419DRAFT_138519 (*gst*), M419DRAFT_137795 (*mfs*). Each gene was presented using its gene ID from the *T. reesei* Rut-C30 genome database (https://www.ncbi.nlm.nih.gov/genome/323?genome_assembly_id=49799). **B** Transcription levels of sugar transporter genes *crt1*, *mfs*, *gst*, and *lac1* in *T*. *reesei* Rut-C30 cultured on TMM + 2% cellulose/glucose at 24 h, 72 h, 120 h, and 168 h. **C** Transmembrane domains of four sugar transporters obtained from the webserver Protter. The green square represents n-glycosylation motif and red circle represents the signal peptide. **D** The (hemi)cellulase activities and protein secretion of *T*. *reesei* Ku70, ΔMFS, ΔGST, and ΔLAC1 cultured in TMM + 2% cellulose for 120 h. **E** The colony diameters and **F** spore amounts of *T*. *reesei* Ku70, ΔMFS, ΔGST, and ΔLAC1 grown on TMM plates with 2% cellulose at 120 h. Values are the mean of three biological replicates and error bars are the standard deviation of these three replicates. Asterisks indicate significant differences (*p < 0.05, **p < 0.01, ***p < 0.001) as assessed by Student’s *t* test

To find sugar transporters related to cellulase production, transcriptome sequencing analysis was performed by comparing Rut-C30 grown on cellulose to the one on glucose. A total of 49 Differentially Expressed Genes (DEGs) were predicted to be sugar transporters for maltose, fucose, xylose, lactose, d-galactonate, and unknown sugars (Fig. 1A and Additional file 1: Table S1). 33 DEGs were upregulated, of which the fold change of gene M419DRAFT_127980 (*lac1*) was highest followed by M419DRAFT_138519 (*gst*), while 16 DEGs were downregulated. In these DEGs, the top three sugar transporter genes with the highest expression level under cellulose condition were M419DRAFT_109243 (*crt1*), M419DRAFT_137795 (*mfs*), and M419DRAFT_138519 (*gst*) according to Fragments Per Kilobase of exon model per Million mapped fragments (FPKM) (Additional file 1: Table S1). Therefore, the expression levels of these four genes (*crt1*, *mfs*, *gst,* and *lac1*) were measured by qRT-PCR in *T*. *reesei* Rut-C30 cultivated on cellulose (Fig. 1B). Gene *crt1* possessed the highest mRNA expression level, followed by *mfs*, *gst*, and *lac1* in a decreasing order. All these four transporter genes exhibited higher mRNA levels in *T. reesei* grown on cellulose than on glucose, marching well the transcriptome data and demonstrating that their increased expression might benefit the cellulase production. As shown by structure analysis, all four sugar transporters belong to MFS transporters. There are 12 transmembrane domains in CRT1 and GST, and 11 in MFS and LAC1 (Fig. 1C) [26]. Sugar transporters CRT1, MFS, and GST have the n-glycosylation motifs, while LAC1 does not. The transporting sugars of MFS and GST are unknown, and CRT1 and LAC1 are putative lactose permeases. Since gene *crt1* essential for cellulase production has been well studied [16, 22, 24, 27], we focused on the other three sugar transporters *mfs*, *gst,* and *lac1* in further study.

Next, sugar transporter genes *mfs*, *gst,* and *lac1* were deleted individually using Ku70 as the parental strain, leading to the knockout strains ΔMFS, ΔGST, and ΔLAC1. The cellulase-producing ability of these recombinant strains were assayed (Fig. 1D and Additional file 2: Fig. S1). The individual deletion of the three sugar transporter genes notably inhibited cellulase production. The FPase activity, CMCase activity, pNPCase activity, pNPGase activity, pNPxase activity, and secreted protein concentration in strain ΔMFS were 1.42 IU/mL, 1.69 IU/mL, 0.29 IU/mL, 0.43 IU/mL, 0.26 IU/mL, and 3.74 mg/mL, only 39.75%, 38.66%, 37.19%, 51.57%, 36.30%, and 54.65% of that in strain Ku70, respectively. The decreased degree of cellulase production in strain ΔGST was similar to strain ΔMFS, while higher inhibition effect was found in strain ΔLAC1 than in strain ΔMFS. The cellulase production was almost abolished in strain ΔLAC1, indicating that it is essential for cellulase production. This is very interesting given that the mRNA abundance of *lac1* was lowest among the four tested sugar transporters (Fig. 1B). It seems that although its mRNA was very low, *lac1* is still indispensable to cellulase production.

In addition, the phenotype of the deletion strains was profiled in terms of cell growth and sporulation ability. The growth of strain Ku70 and deletion strains was investigated by measuring colony diameters on TMM + 2% cellulose plates (Fig. 1E). The colony diameters of the recombinant strains were smaller than that of Ku70 with strain ΔLAC1 possessing the smallest colony diameter. The sporulation ability of the three deletion strains was decreased notably (Fig. 1F). The lowest sporulation amount was found in strain ΔLAC1, only 26.1% of that in Ku70. Taken together, knocking-out genes encoding sugar transporters *mfs*, *gst,* and *lac1* impaired cellulase production, cell growth, and the sporulation ability in *T. reesei* Ku70 with *lac1* deletion being the worse. Particularly, it seems that LAC1 is required for cellulase production in Ku70 on cellulose.

### The impact of the three sugar transporters on sugar consumption and biomass formation

**Fig. 2:**
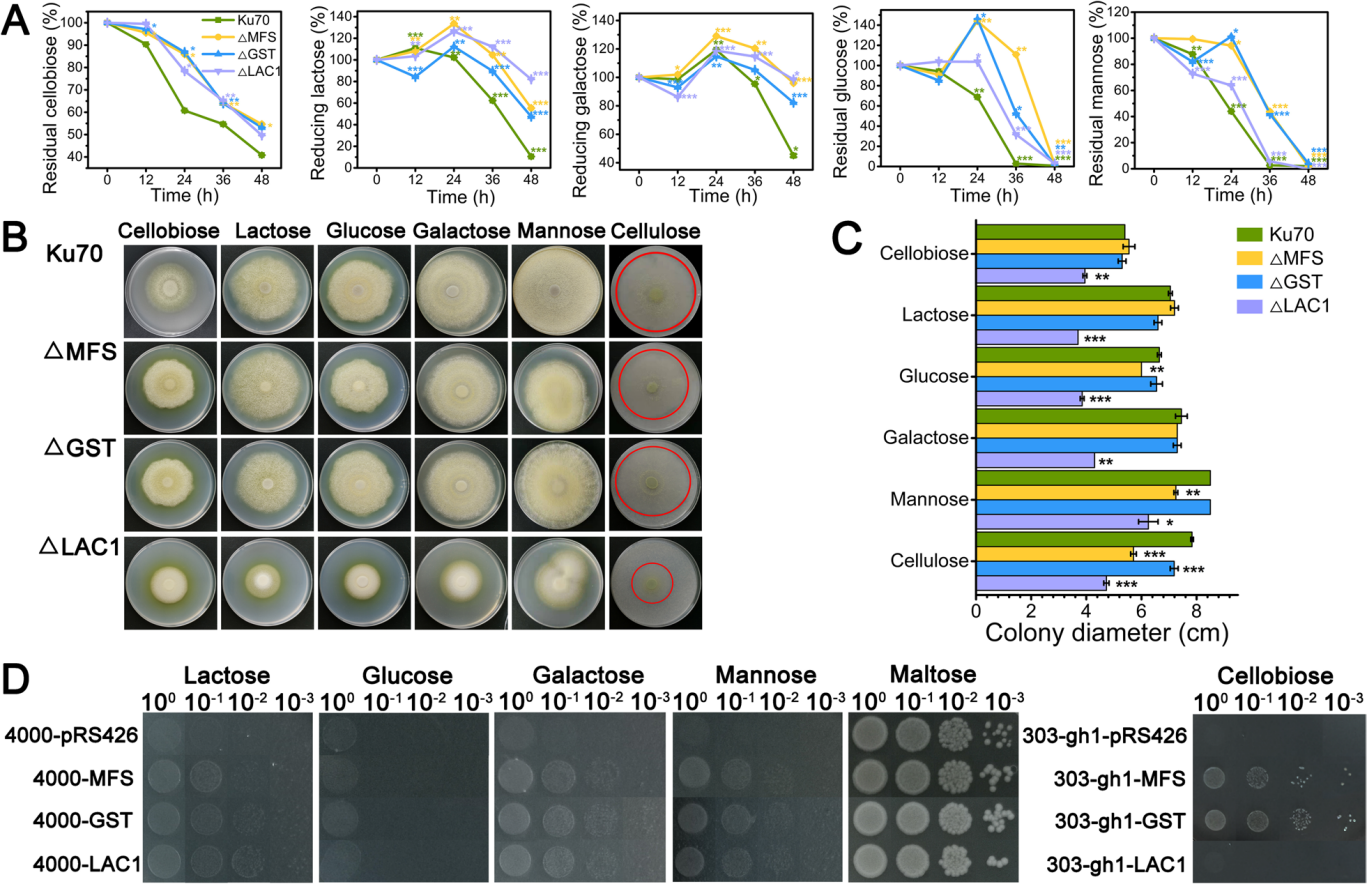
The effect of the three sugar transporters on sugar transport and biomass accumulation. **A** HPLC analysis of residual sugars in the supernatant of *T. reesei* Ku70, ΔMFS, ΔGST, and ΔLAC1 cultured on 1% cellobiose + 50 μg/mL 1-deoxynojirimycin, 1% lactose, 1% glucose, 1% galactose, or 1% mannose. **B** Growth of *T. reesei* Ku70, ΔMFS, ΔGST, and ΔLAC1 on different sugars for 168 h. **C** The corresponding colony diameters in (**B**) were calculated. Values are the mean of three biological replicates and error bars are the standard deviation of these three replicates. **D** Growth analysis of *S. cerevisiae* 303-gh1-pRS426, 303-gh1-MFS, 303-gh1-GST, and 303-gh1-LAC1 in the presence of 1% cellobiose and *S. cerevisiae* 4000-pRS426, 4000-MFS, 4000-GST, and 4000-LAC1 in the presence of 2% maltose, 1% lactose, 1% glucose, 1% galactose, or 1% mannose. Asterisks indicate significant differences (*p < 0.05, **p < 0.01, ***p < 0.001) as assessed by Student’s *t* test

We assessed the sugar uptake ability of the knockout strains ΔMFS, ΔGST, and ΔLAC1 on disaccharides (cellobiose and lactose) and monosaccharides (glucose, galactose, and mannose). As shown in Fig. 2A, we found that the consumption of all the tested sugars by strains ΔMFS, ΔGST, and ΔLAC1 was delayed to varying degrees compared with strain Ku70. Cellobiose, galactose, and lactose were not fully absorbed within 48 h in all *T. reesei* strains. The utilization of cellobiose was noticeably repressed in all the three deletion strains. Deletion of gene *lac1* inhibited most the utilization of lactose, followed by genes *mfs* and *gst,* which is reasonable given that LAC1 is predicted to be lactose permease. The residual glucose in the supernatant of Ku70 was decreased gradually to zero in 36 h, while that in strains ΔMFS, ΔGST, and ΔLAC1 was not reduced significantly in 24 h, but fell sharply from 24 to 48 h. On mannose, a similar pattern to that on cellobiose or glucose was observed in strains ΔMFS and ΔGST but not in ΔLAC1. The mannose uptake of strain ΔLAC1 was comparable to strain Ku70. It seems that the transporters MFS, GST, and LAC1 are involved in the cellular uptake and/or utilization of all the tested sugars (cellobiose, lactose, glucose, galactose, and mannose), except that LAC1 might not be related to that of mannose. The recovery of sugar internalization after 24 h inhibition indicated that there might be functional redundancy proteins for these sugar transporters in *T. reesei*. The presence of these sugar transporters enables *T. reesei* to use sugar in a rapid way.

Moreover, the colony diameters of strains *T*. *reesei* Ku70, ΔMFS, ΔGST, and ΔLAC1 were calculated on solid medium containing 1% (w/v) cellobiose, lactose, glucose, galactose, mannose, or cellulose (Fig. 2B and C). The radial growth of the mutant strains ΔMFS and ΔGST did not exhibit significant difference in the presence of respective tested sugar. Nevertheless, the colony diameter of strain ΔLAC1 was noticeably decreased in comparison to that of Ku70 on all tested sugar, indicating that the absence of *lac1* severely retarded the biomass accumulation in *T. reesei*.

To further investigate their sugar transport function, genes *mfs*, *gst*, and *lac1* were heterologously expressed in *S. cerevisiae* EBY.VW4000 to obtain recombinant strains 4000-MFS, 4000-GST, and 4000-LAC1, respectively. The empty plasmid pRS426 was also transformed into *S. cerevisiae* EBY.VW4000 to get the control strain 4000-pRS426. The mutant strains were spotted on SC-Ura^−^ plates supplemented with 1% lactose, 1% glucose, 1% galactose, or 1% mannose (Fig. 2D). Maltose is the only carbon source that *S. cerevisiae* EBY.VW4000 can used for growth; thus, the mutant strains grew best on the plate containing maltose and showed insignificant difference between each other. Meanwhile, all the strains did not grow out on glucose-containing plates. Strains 4000-pRS426 were unable to grow on lactose, galactose, and mannose, while strains 4000-MFS, 4000-GST, and 4000-LAC1 could utilize these sugars to grow in varying degrees. However, the mutant strains were not grown very well, indicating the transport ability of MFS, GST, and LAC1 for these sugars were very weak. Moreover, since *S. cerevisiae* cannot assimilate cellobiose, *β*-glucosidase gene *gh1-1* and genes *mfs*, *gst*, and *lac1* were co-expressed in *S. cerevisiae* W303 to obtain recombinant strains 303-gh1-MFS, 303-gh1-GST, and 303-gh1-LAC1, respectively. In the same way, we also obtained control strain 303-gh1-pRS426 by transforming the empty plasmid pRS426 into *S. cerevisiae* W303. Both strain 303-gh1-pRS426 and strain 303-gh1-LAC1 were not grown out on cellobiose, while strains 303-gh1-MFS and 303-gh1-GST were grown well on cellobiose. Altogether, sugar transporters MFS, GST, and LAC1 did not transport glucose or maltose, and might weakly transport lactose, galactose, and mannose. Only sugar transporters MFS and GST can transport cellobiose, while sugar transporter LAC1 cannot.

Taken together, the absence of sugar transporters delayed but not compromised sugar uptake in *T. reesei*. All three sugar transporters displayed weak sugar transporting ability toward cellobiose, lactose, galactose, and mannose, and did not transport glucose and mannose. The deletion of *lac1* inhabits significantly biomass formation of *T. reesei* regardless of sugar type, while the absence of *mfs* and *gst* did not. These results demonstrate that the three sugar transporters facilitate sugar consumption, though they possessed low sugar transporting ability, and *lac1* plays a highly positive role in biomass formation on different sugars.

### Cellular distribution of the sugar transporters

**Fig. 3:**
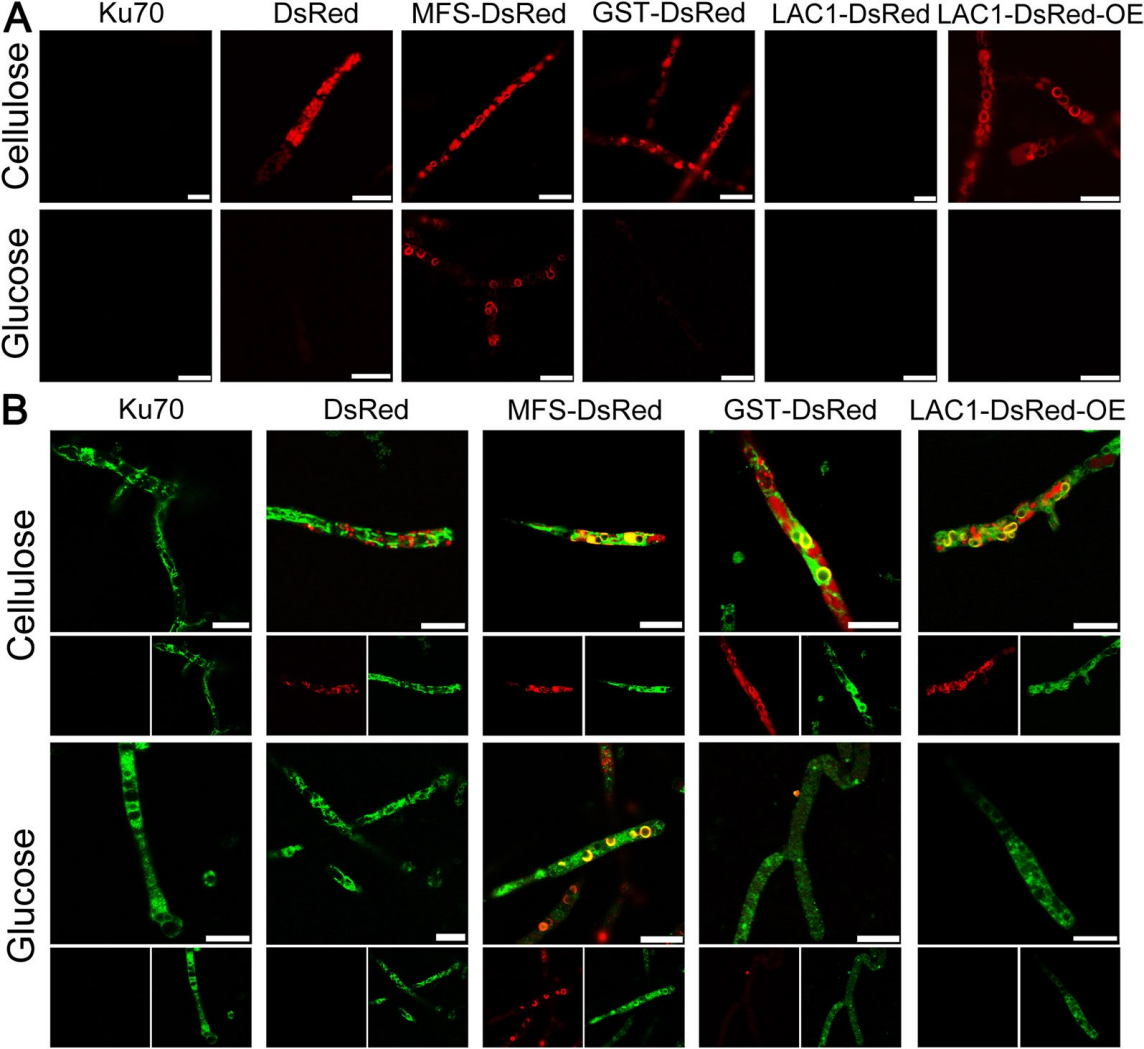
**A** Cellular localization of the three sugar transporters in *T. reesei* cultured in TMM + 2% cellulose at 120 h, respectively. **B** Confocal images of strains Ku70, DsRed, MFS-DsRed, GST-DsRed, and LAC1-DsRed-OE with the treatment of ER-Tracker (Green). Scale bar = 10 μm

We further studied the cellular distribution of the sugar transporters under the control of their own promoters by integrating fluorescence gene DsRed to their 3′-UTR through homologous recombination in *T*. *reesei* Ku70 (Additional file 3: Fig. S2), leading to recombinant strains MFS-DsRed, GST-DsRed, and LAC1-DsRed that expressed DsRed-tagged fusion proteins MFS-DsRed, GST-DsRed, and LAC1-DsRed, respectively. By confocal laser scanning microscopy (CLSM), strong red fluorescence was observed in strain MFS-DsRed grown on cellulose or glucose (Fig. 3A), featuring as dispersed cortical puncta and the characteristic perinuclear ER rings that are often seen in fungi [28–32]. Although it was not that significant, the distribution of MFS-DsRed on the cell membrane was found (Additional file 4: Fig. S3), which is consistent with the structure analysis that MFS contains the signal peptide (Fig. 1C). Red fluorescence was also found in strain GST-DsRed on cellulose with weaker fluorescence intensity than that in strain MFS-DsRed. The fluorescence intensity of strain GST-DsRed grown on glucose was too weak to be observed. Unfortunately, no red fluorescence was detected in strain LAC1-DsRed on cellulose or lactose, possibly owing to the low mRNA level of *lac1* (Fig. 1B). Therefore, LAC1-DsRED was overexpressed under the strong promoter CBH to investigate cellular distribution of LAC1, resulting in the recombinant strain LAC1-DsRED-OE that displayed almost the same (hemi)cellulose activities to the parental strain C30 (Additional file 5: Fig. S4). Strain LAC1-DsRed-OE also showed red fluorescence with the structure of ER rings as found in strain MFS-DsRed and GST-DsRed (Fig. 3). All sugar transporters were not accumulated at apical regions (Additional file 6: Fig. S5). No ER ring was found in in the control strain DsRed expressing red fluorescence protein alone. To further verify that all three sugar transporters are localized in ER, mutant strains MFS-DsRed, GST- DsRed, and LAC1-DsRed were treated with ER-tracker (Fig. 3B). The co-localization of all three sugar transporters MFS-DsRed, GST-DsRed, and LAC1-DsRed with ER was observed, as indicated by the observation of yellow florescence. On the contrary, when treated with ER-tracker, no yellow fluorescence was observed strain DsRed. In short, these three sugar transporters are intracellular sugar transporters, being localized in ER.

### The effect of gene lac1 on the transcriptome of T. reesei

**Fig. 4:**
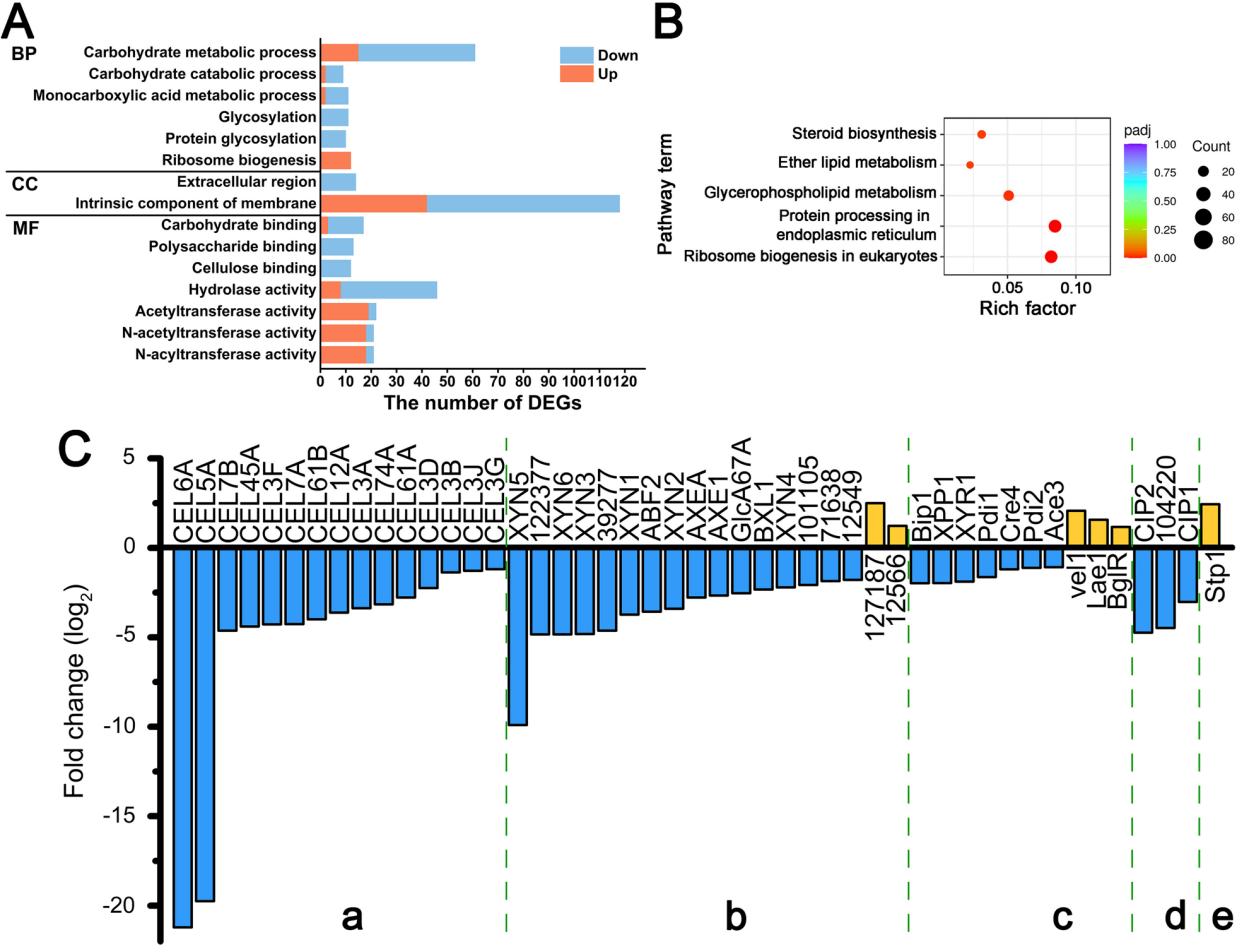
Transcriptome analysis of strain ΔLAC1 grown on TMM + 2% cellulose for 72 h. **A** Gene ontology (GO) functional enrichment analysis of DEGs in strain ΔLAC1. The y axis represents the name of the most enriched GOs that belong to different categories. BP: biological process; CC: cellular component; MF: molecular function. **B** Kyoto Encyclopedia of Genes and Genomes (KEGG) enrichment analysis of DEGs. The y axis represents the name of the most significant enriched pathways. **C** DEGs related to cellulase production, including genes encoding **a** cellulase, **b** hemicellulase, **c** transcriptional factors, **d** nonenzymatic cellulose attacking enzymes, and **e** sugar transporter

To explore the role of *lac1* in cellulase production, we performed the transcriptome analysis of strain ΔLAC1 cultured on cellulose at 72 h. Compared with strain Ku70, there are 2471 deferentially expressed genes (DEGs) in strain ΔLAC1, of which 1454 genes were downregulated and 1017 were upregulated (Additional file 7: Table S2). Significant gene ontology (GO) functional enrichment analysis of these DEGs in strain ΔLAC1 demonstrated that the most enriched biological process (BP) was “carbohydrate metabolic process,” which includes “carbohydrate catabolic process” and “monocarboxylic acid biosynthetic process” (Fig. 4A). The other enriched biological processes belong to “ribosome biogenesis” and “protein glycosylation.” In the category of cellular components (CC), DEGs were concentrated in “extracellular region” and “intrinsic component of membrane.” For the enriched molecular function, “hydrolase activity” has the maximum DEGs, followed by “acetyltransferase activity” and “carbohydrate binding.” All DEGs in “polysaccharide binding” and “cellulose binding,” which are the subcategories of “carbohydrate binding,” were downregulated. By Kyoto Encyclopedia of Genes and Genomes (KEGG) pathway enrichment analysis of DEGs, we screened the top 5 KEGG pathways with padj-value  < 0.05, including “ribosome biogenesis in eukaryotes,” “protein processing in endoplasmic reticulum,” “glycerophospholipid metabolism,” “ether lipid metabolism,” and “steroid biosynthesis” (Fig. 4B). 27 of 30 DEGs in “protein processing in endoplasmic reticulum” were downregulated (Additional file 8: Table S3), and 28 of 30 DEGs in “glycerophospholipid metabolism,” “ether lipid metabolism,” and “steroid biosynthesis” were downregulated except genes M419DRAFT_72858 and M419DRAFT_25484 (Additional file 9: Table S4). On the contrary, the DEGs in “ribosome biogenesis in eukaryotes” were all upregulated (Additional file 10: Table S5). To summarize, the absence of gene *lac1* had impact on cellulase synthesis, ribosome biogenesis, protein glycosylation, lipid metabolism, and acetylation.

A total of 47 DEGs were known or predicted to be related to (hemi)cellulase production in *T. reesei* (Fig. 4C and Additional file 11: Table S6). Most of these DEGs were downregulated, except that genes M419DRAFT_127187, M419DRAFT_12566, *vel1*, *lae1*, *bglR*, and *stp1* (Fig. 4C and Additional file 11: Table S6) were upregulated. Particularly, the transcriptional levels of (hemi)cellulase genes, including 2 cellobiohydrolases (*cel6a* and *cel7a*), 7 endoglucanases (*cel5a*, *cel7b*, *cel45a*, *cel61b*, *cel12a*, *cel74a*, and *cel61a*), 6 *β*-glucosidases (*cel3f*, *cel3a*, *cel3d*, *cel3b*, *cel3j*, and *cel3g*), 6 xylanases (*xyn5*, *xyn6*, *xyn3*, *xyn1*, *xyn2*, and *xyn4*), and 1 *β*-xylosidase (*bxl1*), were all reduced markedly, matching well with the decreased (hemi)cellulase activities as observed above. Genes encoding the auxiliary proteins, like the cellulose-induced proteins CIP1 and CIP2 [33], which have been reported to enhance cellulose degradation, were also down-expressed markedly. Furthermore, 7 cellulase transcription factors were significantly downregulated, including the well-known cellulase transcription activators XYR1 [34] and ACE3 [35]. The transcriptional level of MFS sugar transporter gene *stp1*, the absence of which was found to enhance the cellulase gene induction, was upregulated [22, 36].

### The mRNA dynamic of crucial genes related to cellulase production in T. reesei ΔLAC1

**Fig. 5:**
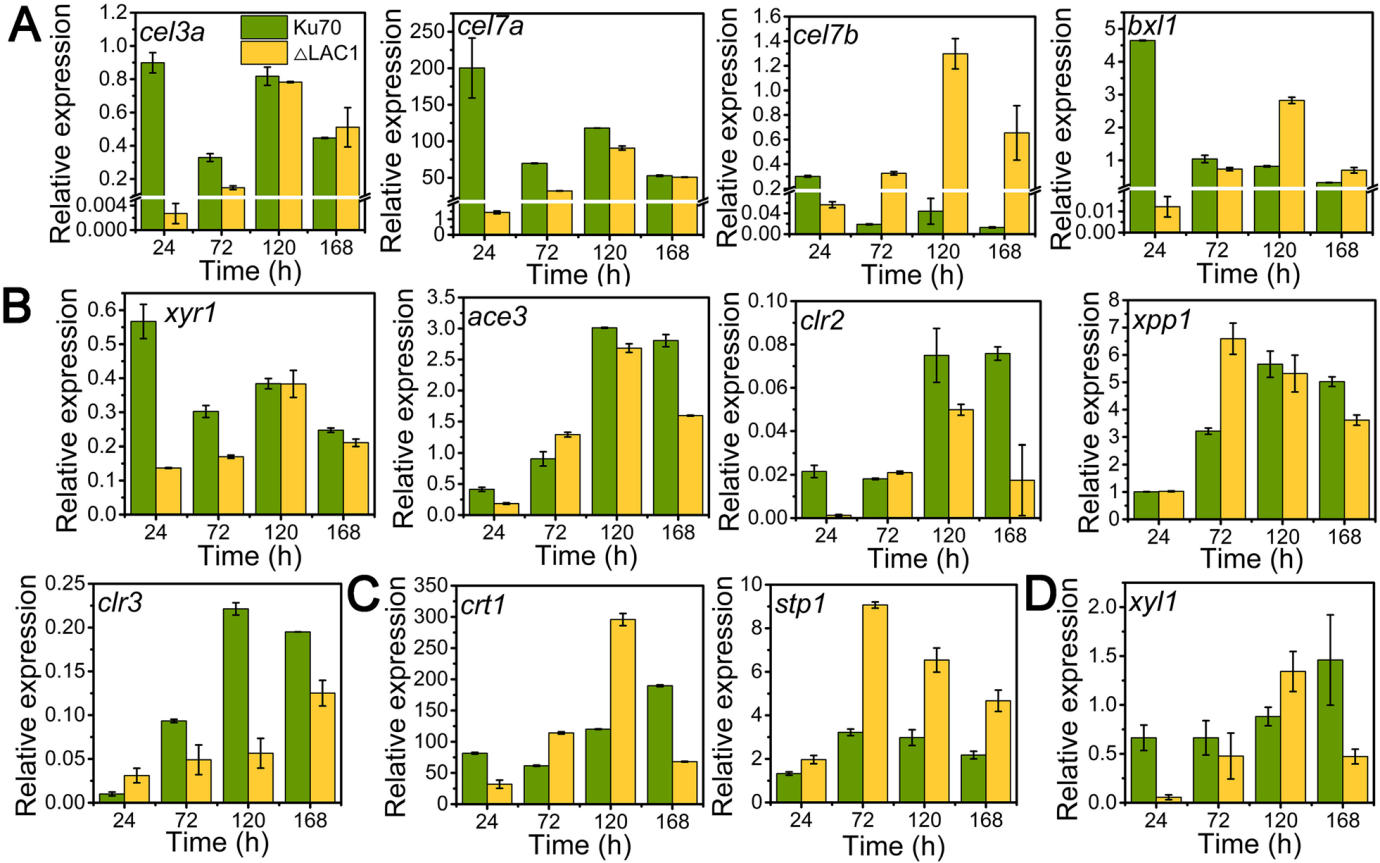
qRT-PCR analysis of the transcript abundance of genes relevant to (hemi)cellulase production in *T. reesei* Ku70 and ΔLAC1 grown on 2% cellulose for 168 h, including genes involved in **A** (hemi)cellulase production, **B** transcription factors, **C** sugar transportation, and **D** lactose metabolism. Values are the mean of three biological replicates and error bars are the standard deviation of these three replicates

We analyzed the relative expression level of genes related to cellulase production, including (hemi)cellulase genes (*cel3a*, *cel7a*, *cel7b*, and *bxl1*), cellulase transcriptional factors (activators *xyr1, ace3*, and *clr2*, repressor *xpp1*, and *clr3*), sugar transporter genes (*crt1* and *stp1*), and lactose metabolism-related gene *xyl1* in *T. reesei* Ku70 and ΔLAC1 during the whole fermentation process for cellulase generation (Fig. 5). The mRNA levels of all these genes were reduced significantly at 24 h in strain ΔLAC1, except genes *clr3*, *xpp1,* and *stp1*. The mRNA levels of (hemi)cellulase genes (*cel3a*, *cel7a*, *cel7b*, and *bxl1*) and the well-known cellulase transcriptional activator XYR1 in ΔLAC1 were all severely reduced at 24 h compared to Ku70, which, however, was recovered to comparable levels to that in Ku70 during the rest fermentation process (Fig. 5A). At 168 h, there was no significant difference in the transcriptional level of genes *cel3a*, *cel7a*, *bxl1*, and *xyr1* between strain Ku70 and strain ΔLAC1, while the expression level of gene *cel7b* of strain ΔLAC1 was much higher than that of strain Ku70. When looking into the data closely, the totally different expression patterns of (hemi)cellulase genes were found in strains Ku70 and ΔLAC1. The relative expression levels of these four genes in strain Ku70 were highest in 24 h and then decreased. However, the expression levels of these four genes of strain ΔLAC1 peaked at 120 h. As compared to strain Ku70, the expression levels of two cellulase transcription activators ACE3 and CLR2 (Fig. 5B), sugar transporter CRT1 (Fig. 5C), and lactose metabolism-related gene *xyl1*(Fig. 5D) in strain ΔLAC1 were reduced at 24 h, were comparable to Ku70 during the middle fermentation stage, and were decreased again at 168 h. The mRNA abundance of gene *clr3* in strain ΔLAC1 was increased at 24 h, but reduced after 24 h. The expression level of sugar transporter STP1 was increased in strain ΔLAC1 during the whole fermentation process, while gene *xpp1* stayed constant in strain ΔLAC1 at 24 h and 120 h, but was much higher than Ku70 at 72 h and lower at 168 h, exhibiting a fluctuated change.

## Discussion

The sensing, uptake, and metabolism of sugar is closely linked with cellulase biosynthesis in cellulolytic fungi grown on cellulose [7–9]. Nevertheless, the identities and functions of the main molecular components of this evolutionarily conserved system are poorly unknown in filamentous fungi. Here, we systematically identified 49 sugar transporters that showed expression difference in *T. reesei* grown on cellulose, demonstrated that they might be associated with cellulase production. Among them, three sugar transporters MFS, GST, and LAC1 exhibited notably increased mRNA level in *T. reesei* grown on cellulose as compared to that on glucose. Moreover, the single deletion of the three sugar transporters noticeably deceased cellulase production, demonstrating they facilitate cellulase synthesis in *T. reesei*. Interestingly, all these three sugar transporters are found in ER, which is out of expectation, as we previously supposed they were on cell membrane according to their structure analysis (Fig. 1C). As far as we know, no intracellular sugar transporter related to cellulase production has been reported. The vast majority of secretory proteins are folded and assembled in ER to achieve their correct tertiary structures [37–39]. All three sugar transporters are distributed in ER where the proper folding, modification, maturation, and secretion of cellulase occur. Through literature search and amino acid sequence analysis, we found some of the analyzed sugar transporters contained one or more ER localization signals (Additional file 12: Fig. S6). There are two ER localization signal sequences reported in the literature: KKXX (Lys-Lys-X-X) [40] which exited in LAC1, and two acid amino acids spaced apart [41] that were found in LAC1 and GST. Interestingly, no ER localization signal sequence was observed in MFS. In addition, although structural analysis demonstrated that they displayed characteristics of sugar transporters, belonging to MFSs, the sugar transporting ability of these three sugar transporters was very weak. Therefore, it is probable that all these sugar transporters are not involved in the cellular uptake of sugar. Instead, they are more likely to take part in the sensing, transport, and/or metabolism of sugars inside cells, like ER. However, the exact function of these three intracellular sugar transporters requires further study.

In particular, the loss of gene *lac1* almost diminished cellulase production, indicating that it is required for *T. reesei* cellulase generation. Up to now, only one sugar transporter CRT1 is reported to be essential for cellulase production in *T. reesei* [16, 22]. Like LAC1, CRT1 belongs to MFS transporter and is considered as lactose permease [6, 22]. Also, the essential role of CRT1 is not related to its sugar transportation ability, which is resembling LAC1 given that LAC1 exhibited very weak sugar transportation capability. Both sugar transporters cannot transport cellobiose when heterologously expressed in *Saccharomyces cerevisiae* [23]. It seems that the essential role of both LAC1 and CRT1 in cellulase production is not related to their sugar transporting ability, although both of them are reported to be lactose permeases. Different from LAC1 residing in cytoplasm, like ER, CRT1 is supposed to be on cell membrane. Furthermore, CRT1 is highly expressed when *T. reesei* is cultivated on wheat straw [42], cellulose [5], and lactose [5]. The mRNA level of *lac1* is much lower than that of *crt1* (Fig. 1B). When *lac1* is deleted, growth deficiency was observed independent on sugar types, implicating that LAC1 is essential for biomass accumulation that is a prerequisite for cellulase production. It is possible that LAC1 inhibited the cellulase production by impairing the cell growth. Whether *lac1* and *crt1* work coordinately or independently to regulate cellulase production is worth exploring in future study.

Transcriptome data showed that *lac1* is important for ER function, including protein glycosylation and lipid synthesis. Most DEGs in strain ΔLAC1 involved in protein processing in ER (Additional file 8: Table S3), protein glycosylation (Additional file 13: Table S7), N-glycan synthesis (Additional file 13: Table S7), sugar transferases (glucosyltransferase, glycosyltransferase, and mannosyltransferase) (Additional file 14: Table S8) were downregulated, showing that LAC1 plays a dominant role in protein n-glycosylation in *T. reesei*. The ER lumen offer a site for numerous glycosylation reactions essential for modifications of proteins and lipids, and for cell wall biosynthesis. These glycosylation reactions require a constant supply of cytosolically synthesized substrates, nucleotide sugars, which are transported by a group of dedicated nucleotide sugar transporters (NST). Considering that LAC1 is localized in ER and predicted to be lactose permease, it is possible that LAC1 is related to the sugar transportation of ER, possibly serving as a sugar transceptor, endomembrane sugar transporter, or nucleotide sugar transporter.

The mRNA dynamic profiling of crucial genes related to cellulase synthesis in strain ΔLAC1 showed that the absence of LAC1 significantly decreased the mRNA level of most genes related to (hemi)cellulase generation except *xpp1*, *clr3*, and *stp1* at 24 h. It seems that LAC1 is important for the initiation of cellulase production, working at the early stage of cellulase production at the transcriptional level. Nevertheless, the transcription level of all these inhibited genes was restored to a similar level to Ku70 after 72 h. Meanwhile, all DEGs associated with ribosome biogenesis were upregulated in strain ΔLAC1 at 72 h (Additional file 10: Table S5). In contrast to the increased mRNA level of (hemi)cellulose genes and the upregulation of genes in ribosome biogenesis, the decreased cellulase production was not recovered (Fig. 1D and Additional file 2: Fig. S1), indicating that the role of *lac1* might work in the post-transcription regulation of cellulase production during the later stage of cellulase production. Moreover, the mRNA level of the well-known cellulase transcriptional activator XYR1 and sugar transporter CRT1 was also recovered after 24 h, implying that the regulation of LAC1 is somewhat independent from the XYR1-CRT1 regulation pathway. The finding that it was *crt1* rather than *lac1* that was deferentially expressed in *T. reesei* with the deletion of *xyr1* further supports this view [11].

Different from the fully inhibited cellulose activities in both LAC1-DsRed and ΔLAC, strains MFS-DsRed and GST-DsRed displayed similar reduction of FPase and CMCase activities to strains ΔMFS and ΔGST, respectively, but much less repression of pNPCase, pNPGase, and pNPxase activities, and protein secretion level than strains ΔMFS and ΔGST, respectively. One possibility for the compromised (hemi)cellulose activities in strains MFS-DsRed and GST-DsRed is that fusion proteins MFS-DsRed and GST-DsRed were not functional. As we observed clear red fluorescence in ER in strains MFS-DsRed and GST-DsRed, which was completely different from the fluorescence pattern in the control strain DsRed where fluorescence protein DsRed was distributed in the cytoplasm rather than ER, we prefer to exclude the possibility that proteins MFS-DsRed and GST-DsRed were not functional. Moreover, ER is the place for protein folding and modification, but not for protein cleavage. The possibility that DsRed-tagged proteins were proteolytically cleaved is small. Also, if DsRed was free from DsRed-tagged protein, it would be exported outside ER to the cytoplasm, as it is well known that DsRed is not ER oriented. Another possibility is that the insertion of the DsRed-labeled cassette before the 3′-UTR of MFS and GST might downregulate their gene expression and then the (hemi)cellulose activities, as in the case of sugar transporter LAC1. Further investigation will be required to explore these possibilities.

## Conclusion

In summary, three sugar transporters MFS, GST, and LAC1 were first discovered to be intracellular sugar transporters localized in ER with weak sugar transporting ability, playing a positive role in sugar utilization and cellulase synthesis in *T. reesei*. In particular, LAC1 was found to be essential for cellulase biosynthesis and biomass formation. The deletion of *lac1* downregulated genes in protein glycosylation in ER and lipid synthesis, implying that LAC1 plays a crucial role in ER function on protein processing and lipid synthesis. LAC1 regulated cellulase production at the transcription level at early fermentation stage, and at post-transcriptional level at late fermentation stage that is independent of CRT1 and XYR1. These findings expand our knowledge on endomembrane sugar transporters and their role in cellulase biosynthesis.

## Materials and methods

### Microbial strains, plasmids, and cultivation conditions

*Escherichia coli* DH5α was used to construct and propagate plasmid. *Agrobacterium tumefaciens* AGL-1 was utilized as a T-DNA donor to mediate fungal transformation. *T. reesei* Rut-C30 (CICC 13052, ATCC 56765) was purchased from China Center of Industrial Culture Collection. *T. reesei* Ku70 [43], *Saccharomyces cerevisiae* strain EBY.VW4000, and plasmid pXBthg were obtained from Professor Wei Wang from East China University of Science and Technology, while plasmid pRS426ADH was a gift from Professor Zhihua Zhou from Key Laboratory of Synthetic Biology, Shanghai [44]. *Saccharomyces cerevisiae* strain W303 and plasmid pRS425ADHgh1-1 were obtained from Weifeng Liu from School of Life Science, Shandong University [22]. *E. coli* DH5α and *A. tumefaciens* AGL1 were cultured in Luria–Bertani (LB) at 37 ℃ and 28 ℃ separately. *S. cerevisiae* EBY.VW4000 and W303 were grown on Yeast Extract Peptone Dextrose Medium (YPD) with 2% maltose at 30 ℃ and its transformants were screened on the Synthetic Complete medium without uracil (SC-Ura^−^) or without uracil and leucine (SC-Ura^−^-Leu^−^). *T. reesei* was cultured in Sabouraud Dextrose Broth (SDB) initially and then transferred onto potato dextrose agar (PDA) plates for conidia production and transferred into Trichoderma medium media (TMM) [45] with different carbon sources for cellulase production. The TMM medium contains the following substances: (NH_4_)_2_SO_4_, 4 g/L; KH_2_PO_4_, 6.5 g/L; Yeast extract, 0.25 g/L; Tryptone, 0.75 g/L; Maleic acid, 11.6 g/L; FeSO_4_ * 7H_2_O, 0.005 g/L; MnSO_4_ * H_2_O, 0.0016 g/L; ZnSO_4_ * 7 H_2_O, 0.0014 g/L; CoCl_2_ * 6H_2_O 0.002 g/L; Urea, 1.00 g/L; CaCl_2_, 0.60 g/L; MgSO_4_, 0.60 g/L; Tween 80, 186 μL/L. The pH of TMM was adjusted to 5.8–6.0 by sodium hydroxide.

### Cultivation of T. reesei in shake flask

Five percent (v/v, 10^7^/mL) conidia of *T*. *reesei* were inoculated into 10 mL SDB and cultured at 28 °C with 200 rpm for 72 h. Then, 5 mL pre-grown mycelia were transferred into 50 mL TMM media with 2% cellulose or 2% glucose and cultivated at 28 ℃with 200 rpm for 168 h. Samples were collected at different time points for (hemi)cellulase activity assay [46–48], confocal observation and organelle staining of mycelia, and RNA-seq analysis.

### Transcriptome analysis of T. reesei grown on cellulose and glucose

*T. reesei* strains were cultured on TMM media with 2% cellulose or 2% glucose for 72 h and then 6 mL samples were taken and centrifuged for 15 min at 8000 × g at 4 ℃. The supernatant was discarded and the sediment was quickly frozen with liquid nitrogen and stored at – 80 ℃ for RNA-seq analysis [46, 49].

### qRT-PCR analysis

Quantitative reverse transcription PCR (qRT-PCR) was performed as described previously [50]. The relative mRNA level was normalized to the housekeeping gene *sar1* [51]. All the primers used are described in Additional file 15: Table S9. Three biological replicates were performed with three technical replicates for each biological replicate.

### Deletion of genes mfs, gst, and lac1 in T. reesei Ku70

The approximately 1.5-kb upstream and downstream sequences of gene *mfs*, *gst,* or *lac1* were individually cloned from the genomic DNA of Ku70, a Rut-C30 derivative by knocking-out gene *ku70* [43] by PCR amplification, and were inserted into the linearized plasmid pXBthg, leading to recombined plasmids pXBthg-lac1, pXBthg-mfs, and pXBthg-gst, respectively. The resulting plasmids were transformed into *T. reesei* Ku70, respectively, by Agrobacterium tumefaciens-mediated transformation (AMT) method [52], leading to recombinants with the deletion of *mfs, gst,* or *lac1*. Hygromycin B was used as the marker to screen recombinants. Genomic DNA was extracted for PCR and sequencing to verify that the gene *mfs*, *gst,* or *lac1* was deleted in respective recombinants. In this way, deletion strains ΔMFS, ΔGST, and ΔLAC1 were obtained. The primers used for amplification and verification are listed in Additional file 15: Table S9.

### Colony diameter measurement and sporulation assay

The conidia of *T*. *reesei* Ku70, ΔMFS, ΔGST, and ΔLAC1 was individually inoculated onto TMM plates containing 2% cellobiose, lactose, glucose, galactose, mannose, or cellulose and then incubated at 28 ℃ for 120 h. The diameters of *T. reesei* colonies at designated time points were measured with the corresponding photos taken. Meanwhile sporulation was collected from the TMM + 2% cellulose plates using 2 mL 0.02% Tween 80. The spores were counted by a hemocytometer under a confocal microscope SP8 with a 20 × objective. Each carbon source has three biological replicates.

### Residual sugar assay in culture supernatants of T. reesei

The recombinant *T. reesei* strains and parental strain Ku70 were cultured in SDB for 48 h. After centrifugation at 8000 × g for 15 min, the mycelium was collected and then was washed with TMM until there was no glucose in the solution. Then the mycelium was transferred to TMM medium containing 1% different sugars and cultured in shake flask at 28 ℃ with 200 rpm. For evaluating cellobiose uptake more accurately, 50 μg/mL 1-deoxynojirimycin, a *β*-glucosidase inhibitor, was added into 50 mL TMM containing 1% cellobiose to inhibit the degradation of cellobiose by *β*-glucosidase. Samples were taken every 12 h. The supernatant of the samples was diluted to a proper concentration and then filtered with a 0.22 μm membrane. The amount of glucose in the supernatant was determined by Glucose assay Kit (RSBio, China) and the amount of other sugars was determined by high-performance liquid chromatography (HPLC) using Aminex HPX-87H column [25].

### Expression of sugar transporters mfs, gst, and lac1 in Saccharomyces cerevisiae

Due to lacking 20 hexose transporters [53], *S. cerevisiae* EBY.VW4000 has become an important tool for characterizing novel sugar transporters in other fungi [54–56], which can exclude the interference of other sugar transporters in fungi. In order to express sugar transporters in *S. cerevisiae* EBY.VW4000, the sequences of gene *mfs*, *gst*, and *lac1* were individually amplified from the genomic DNA of Rut-C30. The PCR product fragments were linked to the plasmid pRS426 which was linearized by restriction endonucleases *EcoRI* and *HindIII,* respectively, resulting in pRS426-MFS, pRS426-GST, and pRS426-LAC1. *S. cerevisiae* EBY.VW4000 was prepared into competent cell using the EX-Yeast Transformation Kit (Cat.NO. ZC135, Beijing Zoman Biotechnology Co., Ltd). According to the protocol, the recombinant plasmids pRS426, pRS426-MFS, pRS426-GST, and pRS426-LAC1 were individually transformed into competent *S. cerevisiae* EBY.VW4000 to obtain recombinant strains 4000-pRS426, 4000-MFS, 4000-GST, and 4000-LAC1. Since *S. cerevisiae* cannot assimilate cellobiose, plasmid pRS425ADHgh1-1 for the expression of *β*-glucosidase [22] and recombinant plasmids pRS426, pRS426-MFS, pRS426-GST, and pRS426-LAC1 were co-transformed into *S. cerevisiae* W303 [22] simultaneously, leading to the recombinant strains 303-gh1-pRS426, 303-gh1-MFS, 303-gh1-GST, and 303-gh1-LAC1. Transformants were selected on the SC-Ura^−^ plates or SC-Ura^−^-Leu^−^ and the possible colonies were further confirmed by PCR. The primers used in this experiment are listed in Additional file 15: Table S9.

### Growth of Saccharomyces cerevisiae strains on solid medium

*S. cerevisiae* strains 4000-MFS, 4000-GST, 4000-LAC1, 4000-pRS426, 303-gh1-MFS, 303-gh1-GST, 303-gh1-LAC1, and 303-gh1-pRS426 were inoculated into SC-Ura^−^ or SC-Ura^−^-Leu^−^ medium containing 2% maltose for 24–48 h at 30 ℃ at 200 rpm. The cells were centrifuged at 4000 × g for 5 min and then washed twice with sterilized water. The cells were re-suspended in water, diluted to a concentration of 1.0 at OD_600_, and used it as stock solution. Then the stock solution was diluted 10^1^-, 10^2^-, and 10^3^-fold in a gradient and 5 μL of each was added onto the SC-Ura^−^ plates or SC-Ura^−^-Leu^−^ plates containing different sugars. These plates were incubated at 30 ℃ for 72–120 h.

### Construction of T. reesei strains MFS-DsRed, GST-DsRed, LAC1-DsRed, and LAC1-DsRed-OE

To tag sugar transporters at N terminal with fluorescence protein DsRed, the upstream (including the promoter and coding sequence) and downstream (including the terminator) fragments of *mfs*, *gst*, and *lac1* cloned from the genomic DNA of *T. reesei* Rut-C30 were ligated to the plasmid pXBred [57] at *Xho*I and *Bam*HI sites, respectively. The resulting expression vectors were transformed into *T. reesei* Ku70 through AMT approach using hygromycin B as a marker, resulting in mutant strains MFS-DsRed, GST-DsRed, and LAC1-DsRed, respectively. Moreover, the coding region of gene *lac1* was amplified from the genomic DNA of *T. reesei* Rut-C30 and ligated to the plasmid p-DsRed [57] at the *Xba*I site using the ClonExpress II one-step cloning Kit (Vazyme Biotech Co., Ltd., Nanjing, China). The resulting plasmid was extracted and transformed into Rut-C30 through AMT method using hygromycin B as a screening marker, acquiring strain LAC1-DsRed-OE. The confirmation of DNA integration in the mutant strains was performed by PCR and sequencing of the PCR product at Sangon Biotech. The primers used for gene amplification and verification are listed in Additional file 15: Table S9.

### Confocal imaging

Confocal images of *T. reesei* strains DsRed [46], MFS-DsRed, GST-DsRed, LAC1-DsRed, and LAC1-DsRed-OE under different experimental conditions were captured under a confocal laser scanning microscopy (CLSM) using a 100 × magnification oil objective (Leica, Germany). The excitation and emission wavelength were 552 nm and 570–700 nm, respectively. When necessary, *T. reesei* strains were washed with HBSS two times and incubated with GC-PEG-cholesterol-FITC [58] or ER-Green (KeyGEN BioTECH Co. Ltd., China) to stain the cell wall or endoplasmic reticulum. The images were taken with an excitation wavelength of 488 nm and an emission wavelength of 500–550 nm.

## Supplementary Information

**Additional file 1. Table S1. **Genes involved in sugar transport in *T.* *reesei* Rut-C30.

**Additional file 2. Figure S1.** The (hemi)cellulase activities and protein secretion of *T.* *reesei* Ku70, ΔMFS, ΔGST, and ΔLAC1 cultured in TMM+2% cellulose at different time points. (A) FPase: the filter paper activity; (B) CMCase: the CMC activity; (C) pNPCase: the CBH activity; (D) pNPGase: the BGL activity; (E) pNPxase: the xylanase activity; (F) protein secretion. Values are the mean of three biological replicates and error bars are the standard deviation of these three replicates.

**Additional file 3. Figure S2.** Schematic illustration of recombinant construction for labeling transporters (MFS, GST, and LAC1) through homologous recombination. Linker, a short sequence linking sugar transporter gene and DsRed gene; TtrpC, *Aspergillus nidulans* trpC terminator; Hyg, hygromycin B phosphotransferase.

**Additional file 4. Figure S3.** Confocal images of strains MFS-DsRed and GST-DsRed stained with GC-PEG-cholesterol-FITC, a green fluorescence dye for cell walls. Strains MFS-DsRed and GST-DsRed were grown on cellulose for 120 h. The white arrow indicates the separation of cell wall and cell membrane. Scale bar = 10μm.

**Additional file 5. Figure S4.** The (hemi)cellulase activities and protein secretion of *T.* *reesei* C30 and LAC1-DsRed-OE cultured in TMM+2% cellulose.

**Additional file 6. Figure S5.** Cellular lactation of MFS-DsRed, GST-DsRed, and LAC1-DsRed-OE at the apical regions of recombinant strains MFS-DsRed, GST-DsRed, and LAC1-DsRed-OE. Scale bar = 10μm.

**Additional file 7. Table S2.** DEGs in ΔLAC1 compared to Ku70.

**Additional file 8. Table S3.** DEGs related to “protein processing in ER.”

**Additional file 9. Table S4.** f DEGs in “glycerophospholipid metabolism,” “ether lipid metabolism,” and “steroid biosynthesis.”

**Additional file 10. Table S5.** DEGs involved in ribosome biogenesis in eukaryotes as indicated by KEGG.

**Additional file 11. Table S6.** DEGs involved in (hemi)cellulase production in *T.* *reesei* ΔLAC1 compared to Ku70.

**Additional file 12. Figure S6.** ER localization signal motif analysis of sugar transporters LAC1, GST, and MFS. The ER localization signal motifs KKXX and (DE)X(DE) are highlighted with blue background and green background, respectively. The amino acids in red background and white character are strictly identical, in red character are similar in a group, and in blue frame are similar across groups.

**Additional file 13. Table S7.** DEGs related to protein glycosylation in *T.* *reesei* ΔLAC1 compared to Ku70.

**Additional file 14. Table S8.** DEGs related to transferase in *T.* *reesei* ΔLAC1 compared to Ku70.

**Additional file 15. Table S9.** Primers used in this article.

## Data Availability

The data sets supporting the conclusions of this article are included in this article and its Additional files 1 and 2.
