# Supplementary material for: Discovery of ER-localized sugar transporters for cellulase production with lac1 being essential
Source: Biotechnol Biofuels Bioprod. 2022 Nov 29;15:132. doi: 10.1186/s13068-022-02230-x (PMC9706901; doi:10.1186/s13068-022-02230-x)
Supplement: Supplementary file 2 — Additional file 2. Figure S1. The (hemi)cellulase activities and protein secretion of T. reesei Ku70, ΔMFS, ΔGST, and ΔLAC1 cultured in TMM+2% cellulose at different time points. (A) FPase: the filter paper activity; (B) CMCase: the CMC activity; (C) pNPCase: the CBH activity; (D) pNPGase: the BGL activity; (E) pNPxase: the xylanase activity; (F) protein secretion. Values are the mean of three biological replicates and error bars are the standard deviation of these three replicates. [file 13068_2022_2230_MOESM2_ESM.docx]

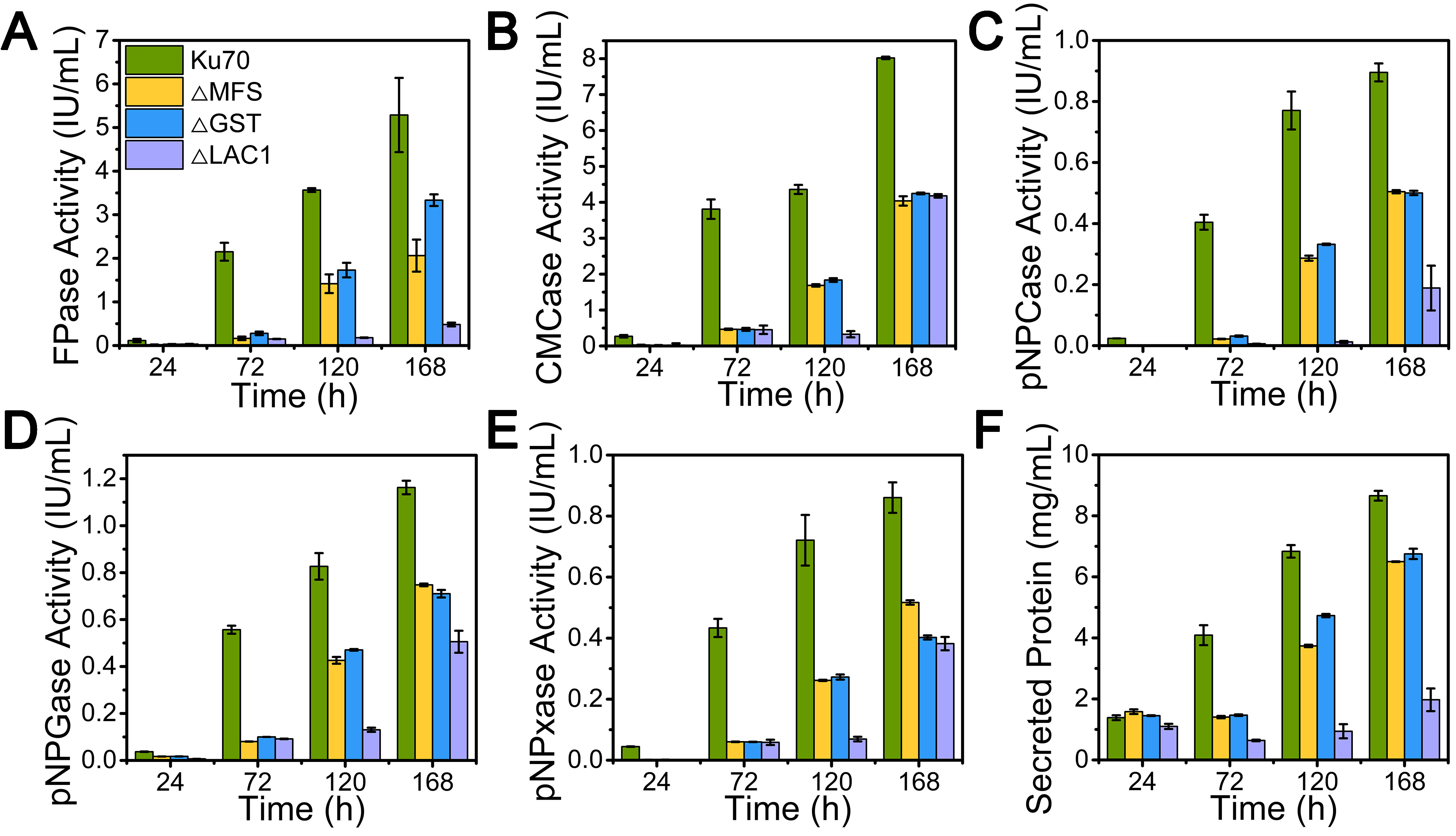


**Additional file 2: Figure S1** The (hemi)cellulase activities and protein secretion of *T.* *reesei* Ku70, ΔMFS, ΔGST, and ΔLAC1 cultured in TMM+2% cellulose at different time points. (A) FPase: the filter paper activity; (B) CMCase: the CMC activity; (C) pNPCase: the CBH activity; (D) pNPGase: the BGL activity; (E) pNPxase: the xylanse activity; (F) protein secretion. Values are the mean of three biological replicates and error bars are the standard deviation of these three replicates.
