# Supplementary material for: Discovery of ER-localized sugar transporters for cellulase production with lac1 being essential
Source: Biotechnol Biofuels Bioprod. 2022 Nov 29;15:132. doi: 10.1186/s13068-022-02230-x (PMC9706901; doi:10.1186/s13068-022-02230-x)
Supplement: Supplementary file 3 — Additional file 3. Figure S2. Schematic illustration of recombinant construction for labeling transporters (MFS, GST, and LAC1) through homologous recombination. Linker, a short sequence linking sugar transporter gene and DsRed gene; TtrpC, Aspergillus nidulans trpC terminator; Hyg, hygromycin B phosphotransferase. [file 13068_2022_2230_MOESM3_ESM.docx]

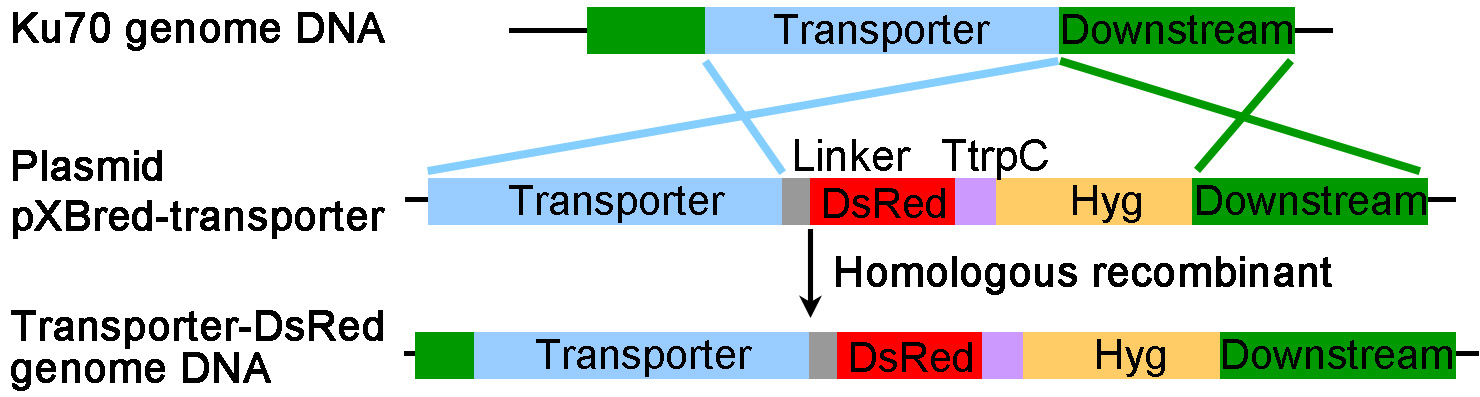


**Additional file 3: Figure S2** Schematic illustration of recombinant construction for labeling transporters (MFS, GST, and LAC1) through homologous recombination. Linker, a short sequence linking sugar transporter gene and DsRed gene; TtrpC, *Aspergillus nidulans* trpC terminator; Hyg, hygromycin B phosphotransferase.
