# Supplementary material for: Discovery of ER-localized sugar transporters for cellulase production with lac1 being essential
Source: Biotechnol Biofuels Bioprod. 2022 Nov 29;15:132. doi: 10.1186/s13068-022-02230-x (PMC9706901; doi:10.1186/s13068-022-02230-x)
Supplement: Supplementary file 4 — Additional file 4. Figure S3. Confocal images of strains MFS-DsRed and GST-DsRed stained with GC-PEG-cholesterol-FITC, a green fluorescence dye for cell walls. Strains MFS-DsRed and GST-DsRed were grown on cellulose for 120 h. The white arrow indicates the separation of cell wall and cell membrane. Scale bar = 10μm. [file 13068_2022_2230_MOESM4_ESM.docx]

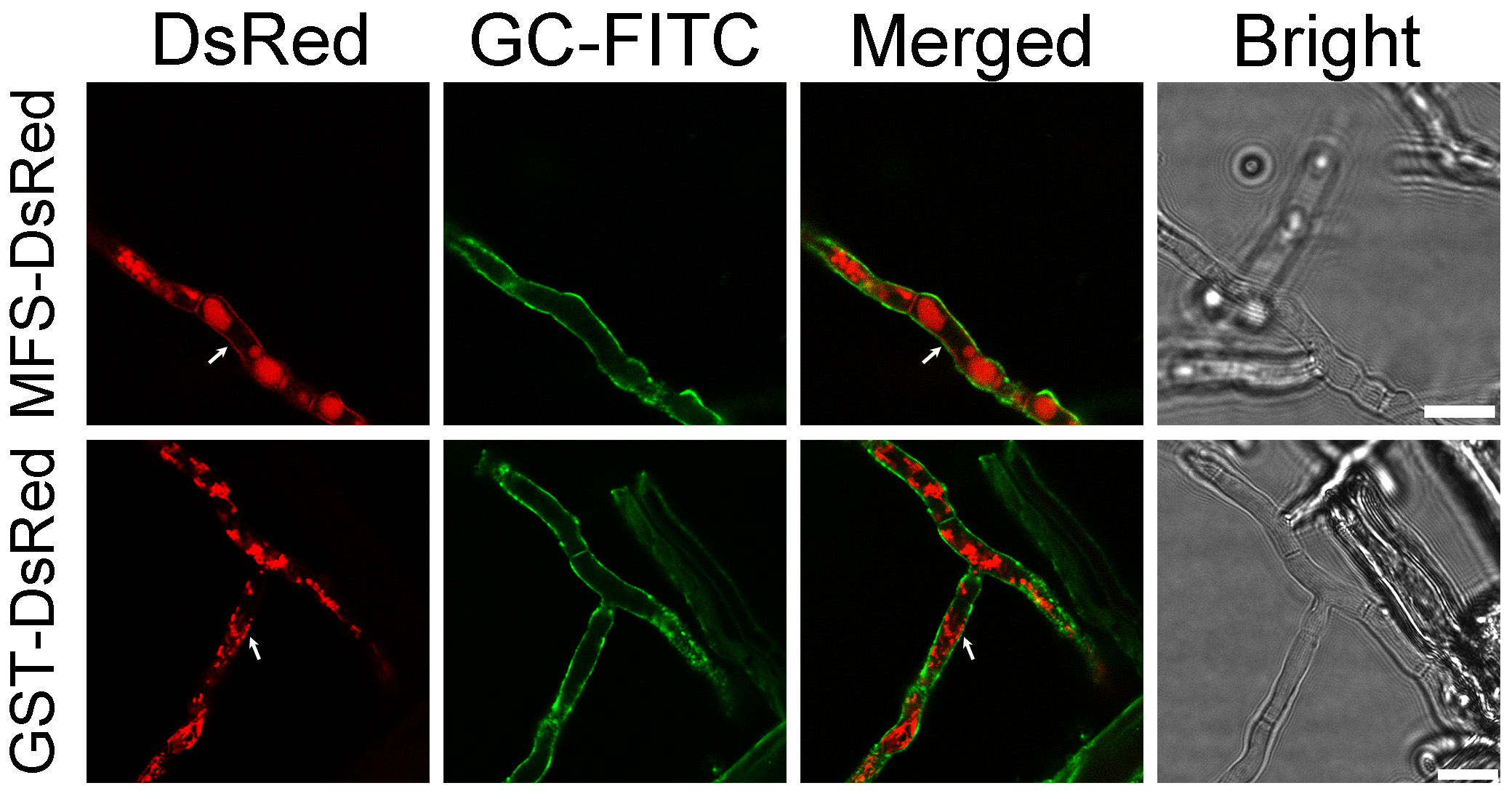


**Additional file 4: Figure S3** Confocal images of strains MFS-DsRed and GST-DsRed stained with GC-PEG-cholesterol-FITC, a green fluorescence dye for cell walls. Strains MFS-DsRed and GST-DsRed were grown on cellulose for 120 h. The white arrow indicates the separation of cell wall and cell membrane. Scale bar = 10 μm.
