# Supplementary material for: Discovery of ER-localized sugar transporters for cellulase production with lac1 being essential
Source: Biotechnol Biofuels Bioprod. 2022 Nov 29;15:132. doi: 10.1186/s13068-022-02230-x (PMC9706901; doi:10.1186/s13068-022-02230-x)
Supplement: Supplementary file 5 — Additional file 5. Figure S4. The (hemi)cellulase activities and protein secretion of T. reesei C30 and LAC1-DsRed-OE cultured in TMM+2% cellulose. [file 13068_2022_2230_MOESM5_ESM.docx]

**
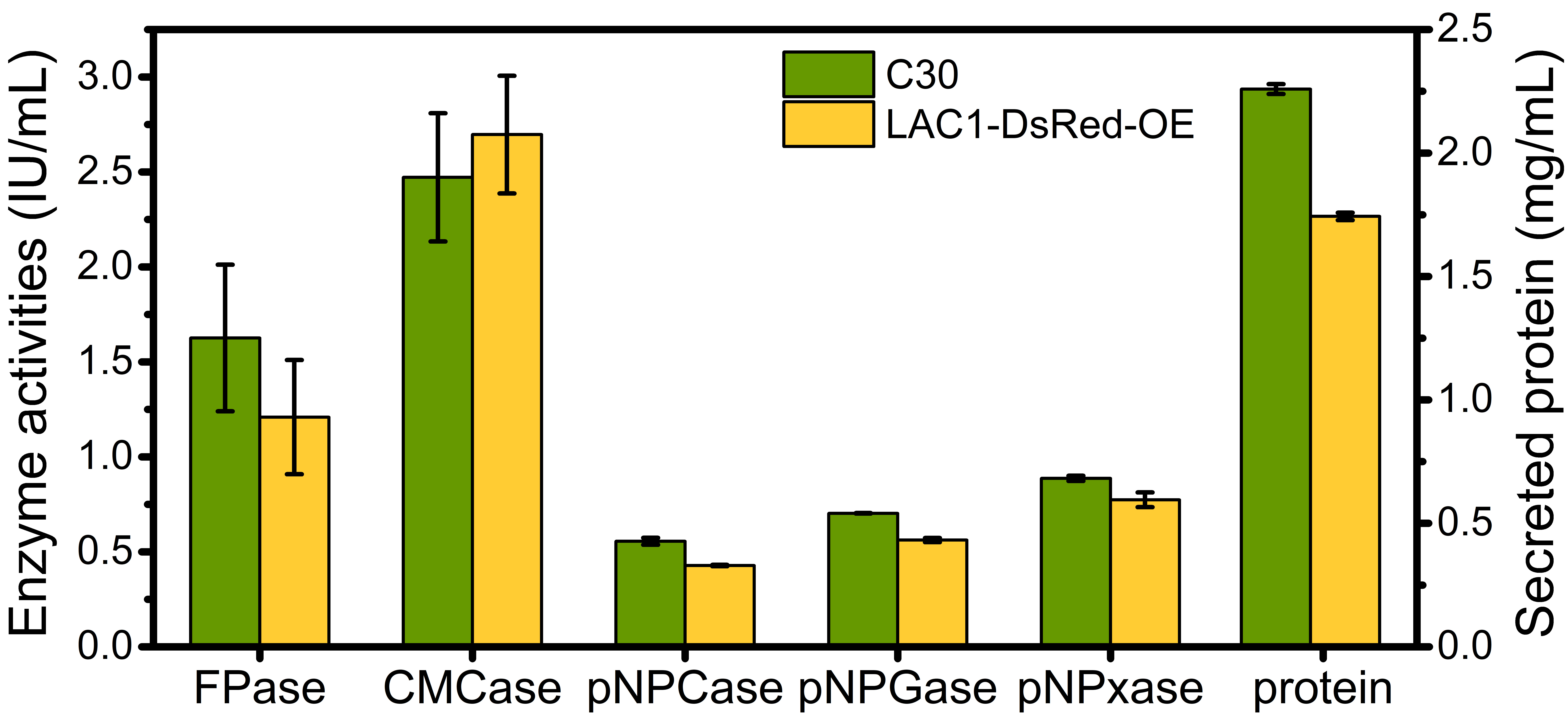
**

**Additional file 5: Figure S4** The (hemi)cellulase activities and protein secretion of *T. reesei* C30 and LAC1-DsRed-OE cultured in TMM+2% cellulose.
