# Supplementary material for: Discovery of ER-localized sugar transporters for cellulase production with lac1 being essential
Source: Biotechnol Biofuels Bioprod. 2022 Nov 29;15:132. doi: 10.1186/s13068-022-02230-x (PMC9706901; doi:10.1186/s13068-022-02230-x)
Supplement: Supplementary file 6 — Additional file 6. Figure S5. Cellular lactation of MFS-DsRed, GST-DsRed, and LAC1-DsRed-OE at the apical regions of recombinant strains MFS-DsRed, GST-DsRed, and LAC1-DsRed-OE. Scale bar = 10μm. [file 13068_2022_2230_MOESM6_ESM.docx]

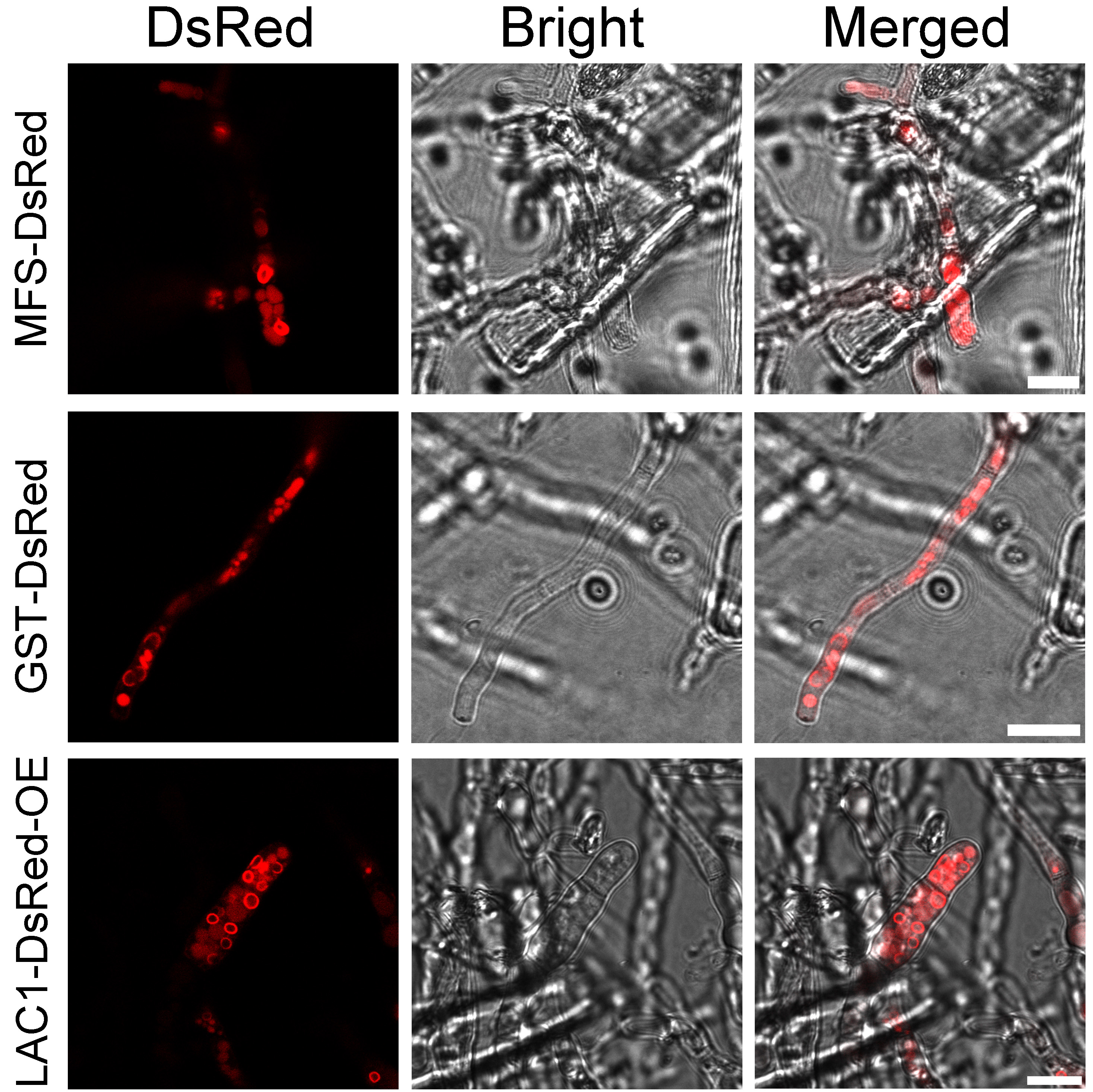


**Additional file 6: Figure S5** Cellular lactation of MFS-DsRed, GST-DsRed, and LAC1-DsRed-OE at the apical regions of recombinant strains MFS-DsRed, GST-DsRed, and LAC1-DsRed-OE. Scale bar = 10μm.
