# Supplementary material for: Discovery of ER-localized sugar transporters for cellulase production with lac1 being essential
Source: Biotechnol Biofuels Bioprod. 2022 Nov 29;15:132. doi: 10.1186/s13068-022-02230-x (PMC9706901; doi:10.1186/s13068-022-02230-x)
Supplement: Supplementary file 12 — Additional file 12. Figure S6. ER localization signal motif analysis of sugar transporters LAC1, GST, and MFS. The ER localization signal motifs KKXX and (DE)X(DE) are highlighted with blue background and green background, respectively. The amino acids in red background and white character are strictly identical, in red character are similar in a group, and in blue frame are similar across groups. [file 13068_2022_2230_MOESM12_ESM.docx]

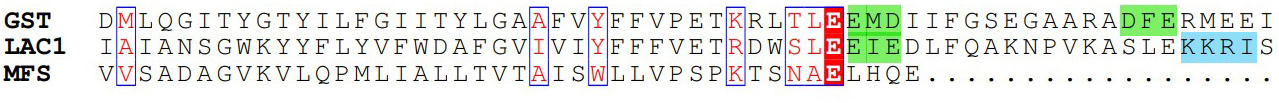


**Additional file 12:** **Figure S6** ER localization signal motif analysis of sugar transporters LAC1, GST, and MFS. The ER localization signal motifs KKXX and (DE)X(DE) were highlighted with blue background and green background respectively. The amino acids in red background and white character are strictly identical, in red character are similar in a group, and in blue frame are similar across groups.
